# Deciphering the Structural Biology of GFAP: Connotations of Its Potency in Presaging the Diagnosis for Traumatic Brain Injury and AD

**DOI:** 10.3390/neurolint17090134

**Published:** 2025-08-26

**Authors:** Sri Harsha Kanuri, Prapthi Jayesh Sirrkay

**Affiliations:** 1 Merit Health Wesley Medical Center, 1501 Hardy Street, Hattiesburg, MS 39402, USA; 2 Medical School, University of Minnesota, Minneapolis, MN 55455, USA

**Keywords:** GFAP, astrogliosis, Alzheimer’s disease, neuronal death, brain atrophy, amyloid beta, tau, neurodegeneration, biomarkers

## Abstract

In Alzheimer’s disease, accumulation of Aβ and tau aggregates in the limbic and cortical regions of the brain forms the pathological basis for the onset of memory loss and cognitive abnormalities. The neuronal desecration inflicted by these toxic pile-ups will rouse the onset of innate immune defense mechanisms including astrogliosis within the neuronal milieu. A potential ramification of astrogliosis is the overproduction and spillage of GFAP into the brain circulation. Execution of GFAP vital physiological functions rests upon the preservation of its filamentous structure as well as its cytoskeletal interactions. Any anomaly that hampers the structural integrity of GFAP will engender filament disassembly, cytoplasmic aggregation, and decreased solubility with the resultant deleterious consequences. The potency of GFAP as a reliable biomarker in the blood also rests on its ability to navigate the glymphatic excretory pathways and spill into the systemic circulation. Recent reports have suggested GFAP is a dependable marker for auguring subtle disease changes in traumatic brain injury (TBI) and AD. However, pathological anomalies such abnormal structural integrity, cleavage, impaired drainage pathways, and alternative isoforms will lessen its potency and thwarts its ability from becoming a full-fledged and stable biomarker for neurological diseases. Understanding the GFAP biology, including factors that influence its structural integrity and excretory pathways, will be crucial and this review underscores these sections in a succinct manner. Thorough comprehension of GFAP biology is the principal step in unearthing its potential as a powerful marker for auguring disease initiation, and progression in TBI and AD.

## 1. Background

### Introduction Section

Alzheimer’s disease (AD) is one of the most important neurodegenerative diseases associated with slow onset and progressive memory impairment. Its incidence as well as prevalence is slowly increasing within the United States and all over the world. A recent report indicated that currently there are around 6.5 million cases of AD in the United States, and this is projected to increase slowly to around 160 million cases by the year 2060 [1]. Dementia is most often misdiagnosed, and thus its exact incidence and prevalence is not clear. On the contrary, another study reported that the age-adjusted prevalence of dementia decreased from 12.2% to 8% from 2000 to 2016 [2]. This decreased prevalence is more apparent in men (40%) as compared to women (20%) [2]. AD is the most common form of dementia, although other forms of dementia such as vascular dementia, frontotemporal dementia, and Lewy body dementia are widely prevalent.

The total costs associated with in-hospital visits, long term care, home care, and hospice care of AD-associated dementia were estimated to be around USD 355 billion in 2021 [1]. Caregiving for AD cases was highly expensive and cost around a billion dollars in the year 2020 alone in the United States.

The most important tell-tale feature of AD is the deposition of amyloid β (Aβ) and neurofibrillary tangles in the limbic and cortical regions of the brain. It has been hypothesized that aberrant processing of the amyloid precursor protein (APP) by β-γ or α-secretases leads to accumulation of Aβ_40_ and Aβ_42_ monomers, which can subsequently transform into senile plaques [3]. The β secretase-mediated degradation pathway predominates over the physiological α-degradation pathway, thus leading to formation of abnormal senile plaques [4]. Aβ aggregates accumulate within vessel walls and neurons provoke deleterious consequences including neuronal death, synaptic dysfunction, blood–brain barrier breakdown, and neuroinflammation [5,6,7].

A staging pattern proposed by Braak et al. was used initially to categorize the progressive accumulation of amyloid deposits within various parts of the brain including the hippocampus and subcortical regions (thalamus, hypothalamus, striatum, cerebellum, subthalamic nucleus, and red nucleus) [8]. The accumulation of neurofibrillary tangles (NFTs) which are formed by misfolded and improperly phosphorylated tau (microtubule-associated protein) are also reported in AD [9,10]. It is also speculated that Aβ aggregates coordinate with NFTs to incite synaptic and neuronal dysfunction [5]. These synaptic and neuronal losses are the underlying factors that eventually lead to thinning of the brain cortex in AD [5]. As the disease progresses gradually from preclinical AD to minimal cognitive impairment (MCI) and finally AD dementia, there is generalized thinning of the frontal, parietal, and temporal lobes, mostly predominant in the left hemisphere [11].

As the disease processes progress, brain immune responses launch a counterattack to minimize this pathological neurological degeneration. As a part of these immune response, astrocyte-induced glial fibrillary acid protein (GFAP) is released to counteract the neuronal damage. This review mainly discusses the biology and functions of GFAP and explores its biomarker function in AD and traumatic brain injury (TBI).

## 2. Methodology

We performed a PubMed search of the relevant articles that enumerate the innate immune responses, particularly astrogliosis and its impact on the neurodegeneration in AD and TBI.

The PubMed search included the peer-reviewed articles (1980–2025) that were published in our topic of interest. Only the PubMed search is included because higher results are retrieved as compared to other databases such as Cochrane. Moreover, there is a limited access to other databases like EMBACE, and CINAH, thus were not included in our search. Moreover, there is a limited access to databases like EMBRACE and CINAH, thus there were not included in our search.

We particularly focused this review on astrogliosis-originated signature GFAP, its structural biology, and its excretory pathways from the brain into the systemic circulation in AD and TBI. This review addresses important structural biology aspects of GFAP including structural components, genetics, functional significance, post-translational modifications, cytoskeletal interactions, and physiological functions. Furthermore, this review summarizes studies from the literature that are focused on the patency of the glymphatic excretory pathways and the journey of GFAP towards the systemic circulation. Once it reaches the blood, the fluctuations in GFAP can relay crucial information regarding underlying subtle brain pathology aberrations in the AD and TBI. This review adds value to the current literature by seamlessly enumerating and linking up the various physiological aspects of GFAP with its ability to function as a stable biomarker in neurodegenerative diseases and TBI.

Our review is a small step in understanding the structural aspects and physiological emigration pathways of GFAP, so that any aberrations secondary to AD and TBI can be comprehended in a pristine manner. The main purpose of this review is to discuss the physiological aspects of GFAP so that its reliability as a biomarker in AD and TBI can be understood. Furthermore, we propound future research studies that would validate its potential for its usage as a reliable biomarker for diagnosis and prognosis of AD and TBI.

## 3. Discussion—Astrocytes in AD

### 3.1. Astrocytes Are a Part of Innate Immune Response in AD

Activated M1 phenotype macrophages as well as Aβ aggregates in the neuronal milieu are documented as activating neighboring astrocytes, whose presence can pave the way for beneficial and deleterious consequences in AD [12,13].

Intracellular events in the astrocytes will induce the upregulation of nitric oxide (NO) synthesis and thence provoke activation of oxidative stress pathways and apoptotic signaling pathways in neurons [14]. In a previous study by DeWitt D.A. et al., macrophages grown in astrocyte-conditioned medium demonstrated a decreased ability to engulf and degrade senile plaques in AD due to inhibitory factors present in the vicinity of neurons [15].

Furthermore, astrocytes are turned on to discharge excitotoxic and inhibitor transmitters namely glutamate and Gamma-aminobutyric acid (GABA) in the neuronal milieu, thus evoking tragic after-effects [16,17]. As a result, impaired synapse conduction, altered glial communication, neuronal cell death, and cognitive disabilities have been reported in AD [13,16,17].

Beta-site APP cleaving enzyme (BACE1) is primarily involved in clearance of Aβ aggregates during AD pathogenesis [18,19]. In a study by Zhou, J. et al., BACE1 expressed in astrocytes modulated the expression of genes that influenced astrocytic uptake and degradation of Aβ aggregates [19]. Mechanistically, BACE1 enhances insulin receptor signaling pathways (P38 MAPK, & ERK1/2 [Extracellular-signal regulated kinase]), and Clusterin (Clu) gene transcription, thus providing the drive for heightened Aβ aggregate clearance [19].

### 3.2. Astrogliosis Is an Innate Immune Response Secondary to Neuronal Death in AD

Astrogliosis is defined as compensatory astrocytic transfiguration secondary to the Aβ-induced neuronal damage in AD [20,21]. This astrocytic switch will promote healing, recovery, and regeneration of damaged neurons in AD [22]. There appears to be a steep rise (4-fold) in the astrocyte assemblage surrounding the senile plaques of AD.

Recent reports have proposed a microbiota–gut–brain axis as an alternative mechanism for developing reactive astrocytes and astrogliosis in the AD brain. According to this speculative hypothesis, genetic predisposition (APOE4 [Apolipoprotein E4]), aging, stress, and substance abuse can induce dysbiosis of microbial flora of the gut [23]. This dysbiosis can thereby enhance the absorption of inflammatory metabolites from the gut into the systemic circulation. These inflammatory metabolites can circumnavigate towards the central nervous system and specifically hamper the blood–brain barrier [BBB] integrity by targeting mitochondrial function [24,25]. BBB derailment would initiate and perpetuate a massive influx of these toxic metabolites, thus triggering neuroinflammation, astrogliosis, and reactive astrocytes [23,25].

A1 astrocytes secrete pro-inflammatory molecules (TNF-α, [Tumor Necrosis Factor 1-Alpha] IL-6 [Interleukin-6], IL-1β [Interleukin-1 beta], and IL-1α [Interleukin-1 alpha]) whereas A2 astrocytes have an anti-inflammatory secretory profile [neurotrophic factors (Prokineticin-2 (PK2), Chitin-like 3, Frizzled class receptor 1, Nrf2, Pentraxin 3 (PTX3), Sphingosine kinase 1, and Transmembrane 4 L6 family member 1] in the AD brain [26,27,28]. A2 astrocytes are likely to promote neuronal survival, growth, synaptogenesis, and phagocytosis. However, the majority of A1 astrocytes present in the AD brain are influential in disease inception and progression through their pro-inflammatory factors [27]. Reactive astrocytes accumulating near the senile plaques are engorged and hypertrophic particularly at the grey–white matter interface of the AD brain [29].

Astrogliosis is characterized by changes in the morphology of astrocytes along with increased expression of markers such as GFAP, CSF YKL-40 [Cerebrospinal fluid chitinase-3 like 1] vimentin-1 and S-100 β [Calcium binding protein beta] [30,31,32]. According to Sofroniew, astrogliosis can be broadly classified into three categories, namely mild, moderate, and severe form. The extreme and severe astrogliosis is characterized by astrocyte proliferation, massive hypertrophy, increased GFAP production, overhanging cellular process, scar formation, and interactions with neighboring neuronal and non-neuronal cell types (peri-vascular fibroblasts, fibrocytes, pericytes, and microglia) [33].

This severe astrogliosis can induce neuronal demise due to overproduction of hydrogen peroxide (H_2_O_2_) and GABA [16,34]. Beneficially, astrogliosis can help to clear the Aβ aggregates from the neuronal interstitium. Astrocytes seem to gravitate towards these Aβ aggregates due to the augmented release of chemoattractants such as MCP-1 [*Monocyte chemoattractant protein-1*] by the injured neurons [35]. Upon reaching these Aβ aggregates, astrocytes secrete various enzymes that can degrade them, thus limiting their stacking up in the brain tissue. On top of that, astrocytes also secrete ApoE2 [Apolipoprotein E-2] proteins which can degrade the Aβ aggregates [13,36]. Zinc-dependent metallo-endopeptidase neprilysin released from the astrocytes also has the potential ability to degrade these accumulating extracellular aggregates in the AD brain [37].

### 3.3. Astrogliosis Is a Primary Source for Assembly and Exudation of GFAP into the Systemic Circulation

Glial fibrillary acid protein (GFAP) is one of the intermediate filaments upregulated during astrogliosis in AD brains [33]. Reports suggest that there is upregulation of GFAP mRNA (Micro Ribonucleic acid] in the sub-pial and sub-ventricular astrocytes bordering the cellular foci of hippocampus in the animal models of AD [38].

Various isoforms of GFAP expressed in the AD mouse models include GFAP-α [Alpha isoform], GFAP-β [Beta isoform], GFAP-γ [Gamma isoform], GFAP-δ [Epsilon isoform], GFAP-κ [Kappa isoform], and GFAP-ζ [Delta isoform] [39].

The cardinal function of GFAP is to provide reinforcement for the astrocyte–BBB framework [40]. One of the important factors that provokes the synthesis of GFAP during astrogliosis is the nitric oxide (NO) overproduction, which occurs under the supervision of lipopolysaccharide (LPS), IFN-γ [Interferon Gamma] and IL-1 β in the neuronal milieu [41]. NO acts upon the downstream targets and energizes the GC [Guanine cyclase]-cGMP [Cyclic GMP]-PKG [Protein kinase G] pathway to instigate the production of GFAP in the astrocytes [41]. Alternatively, other inducers of GFAP production in the astrocytes can include thyroid hormones, glucocorticoids, Transforming growth factor beta 1 [TGF-β1], and glial-derived neurotrophic factor (GDNF) [42].

Once GFAP is produced within the astrocytes, it will be secreted into the extracellular space in the neuronal milieu. From here, it will make its way towards brain parenchyma by navigating through peri-arterial pathways [43]. Next, it enters the capillary circulation, a place where CSF and interstitial fluid inter-exchange flourishes [43]. Subsequently, it will pass through peri-venous pathways and lymphatic circulation, after which it finally makes its way into the systemic circulation [43].

## 4. Physiological Facets of GFAP

### 4.1. What Is This GFAP?

GFAP is one of the main intermediate filaments of the astrocyte cytoskeleton initially identified by Eng et al. in 1971 [44]. Eng et al. isolated this acidic protein from the white matter of patients with multiple sclerosis [44]. It is primarily located within the cytoplasm and has a molecular weight of 50 kDa (Kilo Daltons).

It is classified as an intermediate filament (IF) based on its size (8–12 nm) which is in between actin microfilaments 6 nm [Nanometers] and microtubules 20 nm [45]. An astrocyte has six classes of IFs and GFAP is classified as class III intermediate filament which also includes vimentin, desmin, and peripherin [45]. Any alterations in the astrocyte morphology and number will necessitate modifications in the expression of IFs.

### 4.2. GFAP Expression

Although it is predominantly expressed in the astrocytes of the brain, relatively lower expression in the systemic tissues is documented as well.

Accordingly, Schwann cells of the peripheral nervous system (PNS), glial cells of the enteric nervous system (ENS), epithelial cells of the lens, epithelial cells of the salivary gland, and neoplastic cells of mullerian origin were also shown to express GFAP [42,46].

### 4.3. GFAP Structure

**Figure 1:**
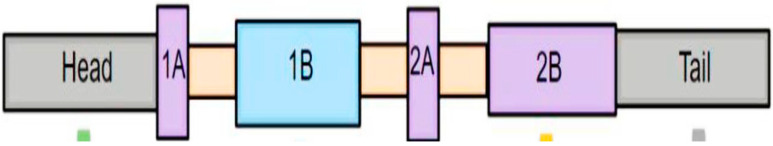
Structure of principal isoform of GFAP. The structure of GFAP can be divided into central alpha helical rod domain and peripheral domains (head and tail). The COOH2 and NH2 domains of the GFAP are mainly responsible for preserving the filament dynamics and functional aspects of GFAP. The central rod domain is further divided into four coils (1A, 1B, 2A, and 2B) which are interlinked with the help of linker domains. The filamentous form of GFAP is sequentially assembled from monomers, dimers, and tetramers. The fully fabricated GFAP is in physiological equilibrium with the GFAP subunits floating in the cytoplasm.

GFAP consists of a central domain and peripheral domains, COOH [Carboxy] and NH2 [N-terminal], respectively (Figure 1) [45]. The amino acid composition of these peripheral COOH and NH2 domains varies among different proteins of class III IF proteins [45]. Reports indicate that COOH and NH2 domains assist in fulfilling different functional aspects of GFAP, namely oligomerization and filament elongation, respectively (Figure 1) [47]. Additionally, the NH2 domain is highly charged and contains five phosphorylation sites whereas the COOH domain is not charged, a feature common to all the class III IF proteins [48].

The linear structure of GFAP is composed of a head domain, rod domain, and tail domain [48]. The central rod domain is formed by four coils (1A, 1B, 2A, and 2B) which are interconnected to each other through linker domains namely 1, 1,2, and 2 [48]. The filamentous form of GFAP is generated by assembly of monomers into dimers and tetramers [48]. These GFAP tetramers further undergo a sequential assembly pathway comprising of ULF formation (unit length filament), longitudinal annealing, and radical compaction [49]. (These three phases of GFAP assembly were primarily regulated by two non-alpha-helical motifs namely MERRRITS-ARRSY and RLSL-RM-PP (RT box motif) which are in the N-terminal domain of GFAP [47]. GFAP mutations form the underlying basis for structural abnormalities, cytoplasmic aggregation, abnormal IF networks, and difficulties in solvent extraction [49]. Furthermore, it seems that assembled GFAP filaments are in kinetic equilibrium with the active GFAP subunits within the cytoplasm during physiological conditions [50]. This translates into the fact that GFAP filaments tento assemble and dissemble at a constant rate where there is a recycling of its subunits at periodic intervals [50]. This GFAP filament subunit exchange might indirectly have some influence on the stability of the astrocyte cytoskeleton and their capability to spring back into action during neuroinflammatory insults [50]. Reports suggest that dynamic assembly and disassembly of individual subunits is regulated by charge of GFAP head domain, which is further modulated by the phosphorylation and dephosphorylation of subunits [51]. This phosphorylation–dephosphorylation seems to happen specifically in the RT box motif, located on the N-terminal domain of GFAP [47]. In transfection experiments of human adenocarcinoma-derived SV13 cell lines, the COOH terminal domain and head domain were primarily responsible for GFAP self-assembly [52]. In parallel to these findings, mutations effecting C-terminal domain of GFAP-induced excessive filament aggregation, decreased solubility, caspase-3 activation, and excessive astrocyte loss, all together forming the underlying pathological basis for manifestation of Alexandria Disease (AxD)) [53].

### 4.4. Physiological Functions of GFAP

As the main intermediate filament of the astrocytes, it plays an important role in various physiological and pathological functions. The cytoskeletal framework and mechanical strength of the astrocytes are the most important and crucial physiological functions of GFAP [54]. Additionally, maintenance of the blood–brain barrier and trophic effect of neurons are mainly dependent on the presence of GFAP [54]. A recent study reported that GFAP is instrumental in facilitating inter-cellular communications between astrocytes and oligodendrocytes for CNS myelination and white matter structural integrity [55]. Important cellular functions of GFAP reported in the literature include motility, migration, proliferation, chaperone-mediated autophagy, synaptic plasticity, glutamine transport, neuronal growth, BBB regulation, injury protection, and myelination [56]. GFAP is demonstrated as interacting with other associated proteins such as plectin, 14-3-3, HSP-27 [Heat Shock protein 27], pre-senilin, and LAMP2A [lysosome-associated membrane protein 2A], thus providing the underlying basis for participating in the crucial physiological functions such as wound healing, mechanical strength, cell cycle regulation, cell fate determination, and autophagy [56].

### 4.5. Post-Translational Modifications of GFAP

The four main post-translational modifications (PTMs) include phosphorylation, acetylation, citrullination, and hyper-palmitoylation.

**Figure 2:**
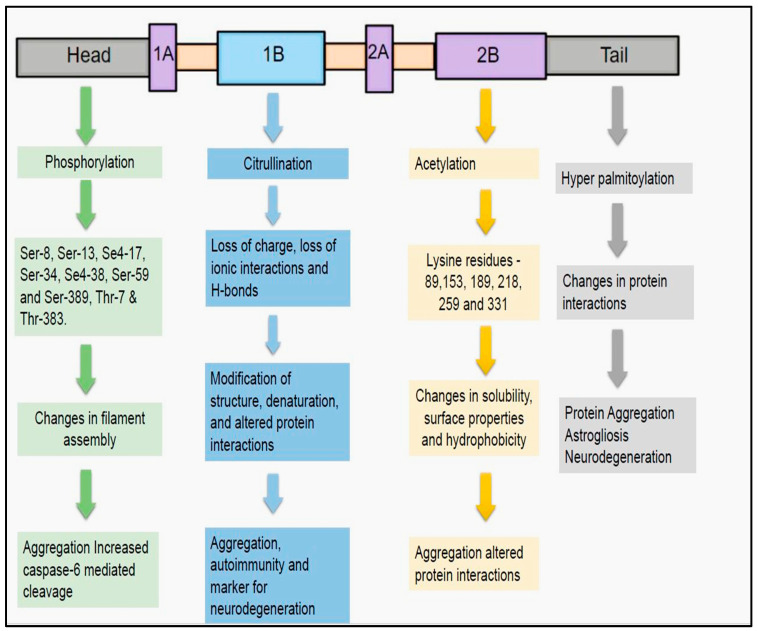
Post-translational modifications of GFAP: The four main post-translational modifications for GFAP include phosphorylation, citrullination, acetylation, and hyper-palmitoylation. These modifications were mainly implicated in revamping the structural assembly, protein interactions, and solubility of GFAP. Any aberrations in these post-translational modifications, can derail the functional characteristics of GFAP. This might result in deleterious consequences including protein aggregation and abnormal cleavage, thus precluding it from being a harbinger for presaging subtle structural changes of neurodegeneration in AD.

Phosphorylation: Phosphorylation is the most important PTM that is mainly implicated in the assembly and disassembly of the GFAP filaments (Figure 2) [42,45]. Assembly of GFAP filaments is mainly dependent on the positive charges of the arginine residues present in the head domain. Phosphorylation of the head domain of the GFAP disrupts these positive charges and thereby results in the disassembly of subunits [51]. As a result, a filamentous structure of GFAP is transformed into a soluble form [57,58,59]. This phenotype change can be apparently reversed by the dephosphorylation of the phospho-GFAP (accomplished by phosphatases), a change that fosters regaining of the polymerized and filamentous structure [57]. Amino-terminal non-alpha-helical head domain of the GFAP filament is mainly involved in this PTM [42,45,51,57]. The crucial phosphorylation sites for GFAP head domain include serine (Ser-8, Ser-13, Se4-17, Ser-34, Se4-38, Ser-59, and Ser-389) and threonine (Thr-7 and Thr-383) residues [42,51]. The main kinases that are involved in the phosphorylation of GFAP head domain can range from Cyclic-AMP [Adenosine monophosphate]-dependent protein kinase A, Ca+2 Calmodulin-dependent kinase II (CAM Kinase II), and Protein kinase C [42,51,56]. Phosphorylation of GFAP seems to play an important role in physiological conditions such as mitosis. Sometimes, phosphorylation of GFAP can be detrimental and can trigger deleterious consequences in some neurodegenerative diseases. In AxD-induced pluripotent.

As pleuripotent stem cell (iPSC)-derived astrocytes formed in AxD, , GFAP ser13 phosphorylation resulted in the peri-nuclear aggregation and bolstered caspase-6-mediated cleavage of GFAP [60].

Citrullination

Another important PTM of GFAP is citrullination (Figure 2). In this modification, arginine amino acids are transformed into citrulline, an enzymatic process catalyzed by peptidyl arginine deiminases (PAD) [61,62]. Due to this PTM, modification of structure, denaturation of protein, and alteration of interaction with neighboring proteins can subsequently occur resulting in the abnormal functioning of the GFAP [62,63]. The underlying reasons for these changes include loss of charge, depletion of ionic interactions, and H-bonds [63]. Citrullinated proteins were notable for inducing autoimmune responses in the Rheumatoid arthritis [64]. In parallel with these findings, anti-GFAP autoantibodies were previously demonstrated in the TBI patients as early as 1–7 days post injury [65]. In this regard, it would be worthwhile to assess the magnitude of GFAP citrullination in these TBI patients to grasp the underlying structural of their autoimmune transpiration. PAD2 expressed primarily in the astrocytes and microglial cells is shown to be activated during neurodegeneration [66]. PAD2-mediated citrullination of GFAP can promote filament disassembly [67]. Evidence of upregulated GFAP citrullination was also previously evidenced in the AD, autoimmune encephalomyelitis (EAE), and cerebral hypoxia [68,69,70]. In AD, activation of PAD2 resulted in the increased accumulation of citrullinated GFAP proteins in the dentate gyrus and stratum radiatum of the hippocampus. It has been hypothesized that increased PAD2 activity as well as hypercalcemia were the main driving forces behind the increased citrullination demonstrated in AD patients [62,70]. Therefore, we can speculate that citrullination can be considered as a harbinger for the onset and rapid progression of disease process in the AD patients [70]. Moreover, these findings adds credence to the hypothesis that therapies aimed at decreasing the activity of PAD2-induced citrullination in the hippocampus might reverse the disease progression and afford better clinical outcomes in the AD patients [70].

Acetylation

Acetylation is the third most important PTM of GFAP (Figure 2). In this PTM, an acetyl group is added to the lysine residue by lysine acetyl transferases and removed by the lysine deacetylases [71]. This PTM results in changes in the solubility, surface properties, and hydrophobicity, all of which can directly instigate aberrations in the protein stability as well as interactions [72]. There were only a few reports where GFAP acetylation was related to the disease pathogenesis of neurodegenerative diseases. In one study, it was demonstrated that the alpha-helical coiled-coil domain of the GFAP filament structure is widely acetylated at positions 89,153, 189, 218, 259, and 331 [73]. The authors attributed this GFAP acetylation to the altered HDAC6 (Histone Deacetylase 6) activity in the astrocytes of the spinal cord [73]. HDAC inhibition in the astrocytes is instrumental in increasing the GFAPδ/GFAPα ratio, thus potentiating the expression of pathogenic GFAP isoform [74]. This altered ratio of GFAP isoforms is the root cause for GFAP aggregation, a tell-tale sign that happens universally in the neurodegenerative diseases such as AxD and AD [74].

Hyper-palmitoylation

S-palmitoylation is an STM characterized by the linking of palmitic acid to cysteine residues of the protein via thioester linkages (Figure 2) [75,76]. The main players for this STM are DHHC palmitoyl transferases, an action that be counteracted by the protein thio-esterases [77,78]. This STM can substantially induce changes in the protein and its neighboring interactions [76]. Previous reports have suggested that this PTM has a role in the pathogenesis of AD. Palmitoylation of the amyloid precursor protein (APP) will instigate its migration to the lipid rafts so that enhanced processing by BACE materializes [79,80]. Strategies aimed at preventing the palmitoylation of APP might be beneficial in reducing the accumulation of Aβ aggregates in AD [81]. In an infantile neuronal ceroid lipofuscinosis (INCL) mouse model, malfunctioning of palmitoyl thioesterases resulted in the increased palmitoylation of GFAP at the cysteine-291 residues [82]. Under these circumstances, changes including increased astrocyte proliferation, astrogliosis, and enhanced neurodegeneration have been documented in the INCL mice [82]. So, strategies to prevent the palmitoylation of GFAP might be beneficial in curbing the disease progression, neuronal death, and neurodegeneration in AD.

### 4.6. How These Modifications Influence Detectability and Interpretation of GFAP Levels in CSF and Plasma

PTM-like ubiquitination might promote the aggregation of GFAP into aggregates known as Rosenthal fibers, thus escaping their detection in the blood [83]. PTMs that are localized to epitope regions might prevent the binding of assay antibodies with GFAP, thus masking its detection in the blood [83]. The cleavage capacity of caspases and calpains can be increased by the presence of phosphorylation PTM. Phosphorylated GFAP can be cleaved by caspase-6 into smaller 38 kDa fragments, which might not be detected by the usual detection methods [83]. Most of the PTMs might influence the stability and structure of GFAP, thereby altering its solubility and half-life of GFAP in the blood. Therefore, all the PTMs might mask the presence of GFAP circulating in the blood, thus leading to inaccurate and reduced detection signals during routine antibody binding assays.

### 4.7. GFAP Cleavage into Subfractions and Its Clinical Significance

**Figure 3:**
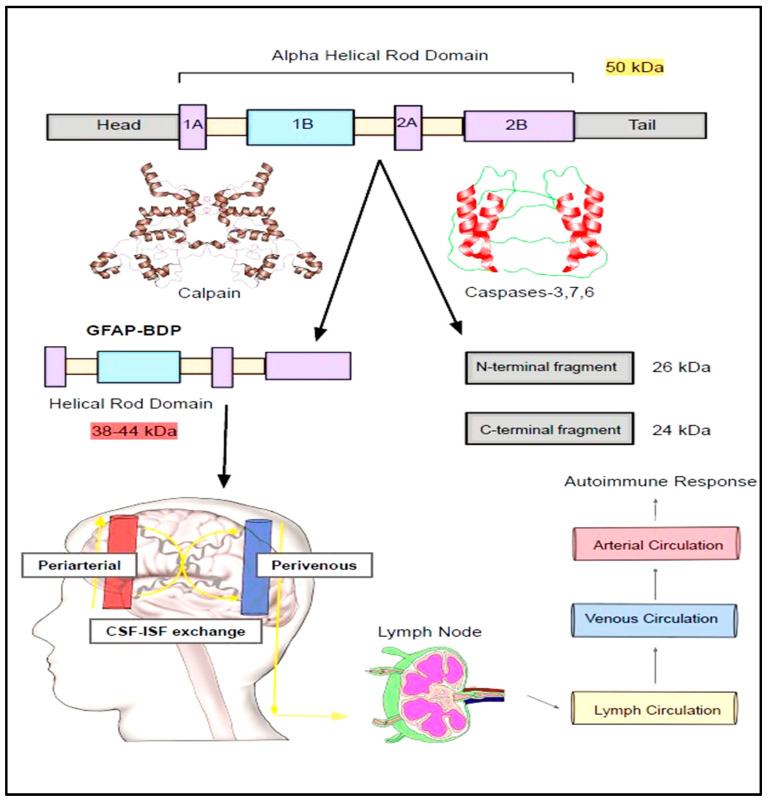
Breakdown down products of GFAP. The principal isoform of GFAP (50 kDa) is vulnerable to being broken down by calpain and caspases into breakdown products (BDPs) of varying molecular weights. These BDPs will then travel along peri-arterial, capillaries, and peri-venous routes and be secreted into lymphatic circulation. These will ultimately be squeezed into the venous circulation and find their way into the systemic circulation. Studies suggest these BDPs were isolated from the CSF of the traumatic brain injury, amyotrophic lateral sclerosis (ALS), motor neurone disease, and spinal cord injury.

**Figure 4:**
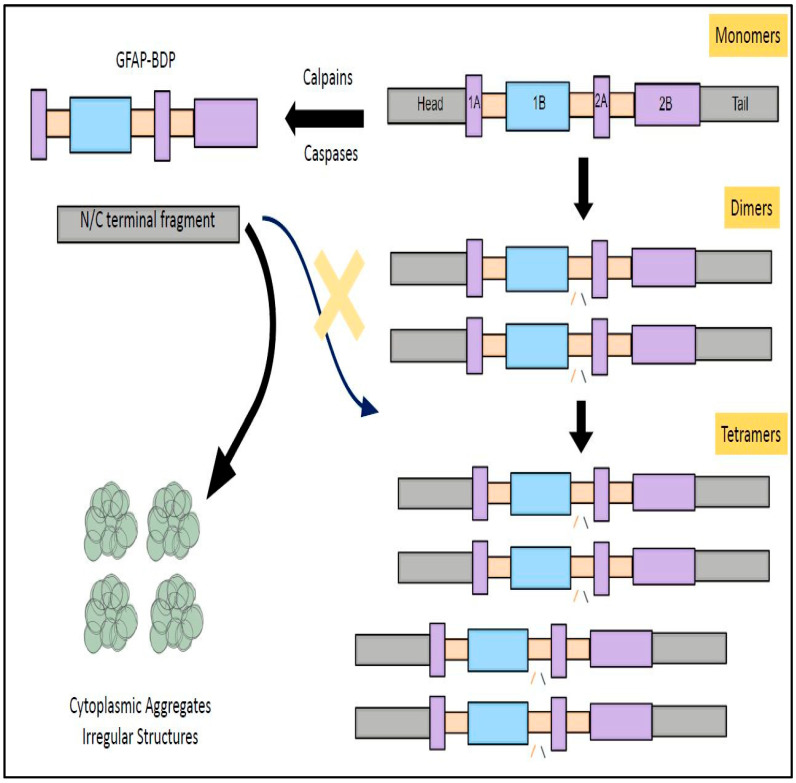
GFAP degradation prevents its polymerization and subverts its physiological functions. The fully functional structure of GFAP (tetramer) is sequentially assembled from monomers and dimers by following a series of steps, namely unit length filament formation, longitudinal annealing, and radical compaction. In instances where GFAP is cleaved by caspases and calpain, this structural assembly process is thwarted. This will result in formation of cytoplasmic aggregates and abrogates its physiological function. These functional aberrations were also shown to form the structural basis for the pathogenesis of Alexandria Disease (AxD).

GFAP can cleaved by calcium activated protease calpain into a truncated peptides known as breakdown products (BDPs) (Figure 3 and Figure 4) [42]. The preferential cleavage sites of calpain in the GFAP include N59-A60 and T383-F384 [42]. According to Yang Z. et al., major N and C terminal cleavage sites of calpain are A-56*A-61 and T-383*Q-388, respectively [84]. Calpain acting on these above-mentioned sites causes removal of N and C-terminal domains while retaining the central rod domain of GFAP resulting in the production of BDPs [42]. Due to calpain-mediated pruning, the molecular weight (MW) GFAP is decreased from its original 50 kDa MW [Molecular weight] size to 38–44 kDa [84,85,86,87]. The generation of these smaller GFAP fragments might provoke an autoimmune response due to the presence of different antigen epitopes.

In line with these findings, the presence of autoimmunity with a 3-7-fold increase in the production of anti-IgG antibodies against these GFAP fragments was noted as early as 0–7 days in the TBI patients [65]. Furthermore, it has been shown that patients who harbor these antibodies for a longer period of time seem to have worse clinical outcomes as compared to those without them [65]. In a clinical research study performed by Papa, L. et al., GFAP-BDP products can be elevated as early as 1–24 h post-traumatic brain injury and were well correlated with the Glasgow Coma Scale, CT (computed tomography) scans, and the need for neurosurgical intervention [88]. The probability of detecting abnormal CT findings in the TBI is significantly higher when plasma values of GFAP-BDP fragments > 0.68 ng/mL [89]. These BDP fragments were isolated from the CSF of rodents and humans with experimental brain injury, TBI, Amyotrophic Lateral Sclerosis (ALS), motor neurone disease, and spinal cord injury (Figure 3 and Figure 4) [85,90,91,92,93].

In addition to calpain, caspases were also alleged to be involved in the breaking down of GFAP into smaller fragments [94,95]. These N- and C-terminal fragments of GFAP generated by cleavage of executioner caspases (Caspase-3 and -7) can be identified by new site-directed caspase cleavage antibodies [93,94]. In mice treated with kainic acid, caspase-3-induced cleavage of GFAP resulted in production of 45 kDa and 40 kDa cleavage fragments [96].

Caspase-6 can split the full length GFAP by acting at four different sites, namely D-78/R-79, D-225/A-226 VELD^225^, and Asp^225^ [84,95]. Preferential action of caspase-6 at Asp^225^ site results in the generation of N-GFAP (MW 26 kDa) and C-GFAP (MW 24 kDa) fragments which differ in their innate ability to facilitate the assembly and disassembly of GFAP sub-units [95]. Specifically, C-GFAP does not have the head and coil-1 domains, thus forfeiting its ability to form filaments [95]. In contrast, N-GFAP is deficient in head and coil-2 domain, and this structural aberration makes it susceptible to form irregular structures and aggregates [95]. Furthermore, caspase-6 cleavage of GFAP and cytoplasmic aggregation was facilitated by phosphorylation of serine-13 amino acid in the GFAP protein [60]. In AD patients, caspase activation and GFAP cleavage in the astrocytes might impair their ability to form robust intercellular communications with neighboring vascular endothelial cells [97,98]. These altered communications might be the harbinger for leaky BBB commonly encountered in the AD patients [97,98].

### 4.8. Cytoskeletal Interactions and Cell Signaling Pathways of GFAP

**Figure 5:**
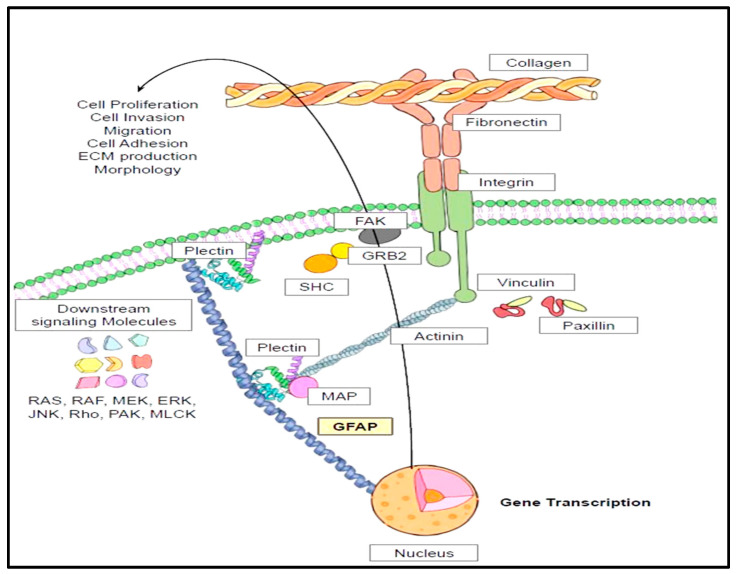
Cytoskeletal interactions of GFAP and its pertinence to its physiological functions. The physiological functions of GFAP in astrocytes include cell survival, proliferation, cell invasion, and migration. These functions are dependent on the cytoskeletal interactions of GFAP with neighboring intermediate filaments including actinin, valin, vinculin, paxillin, and talin. These intermediate filaments are intertwined with extracellular matrix proteins such as fibronectin and laminin and collagen. Engagement of these proteins will lead to activation of downstream signaling mechanisms which forms the underlying structural basis of the crucial physiological functions of GFAP.

Intermediate filaments such as GFAP span through the cytoplasmic space to bridge the gap between the cytoplasmic membrane and nuclear membrane. These intermediate filaments are connected to actin microfilaments though intermediate filament-associated proteins (IFAPs), namely plectins [45,99]. These plectins are interlinked with actin cytoskeleton via actin binding proteins (ABPs) such as actinin, valin, vinculin, paxillin, and talin [45] (Figure 5). So, ABPs are directly connected with integrins which are transmembrane proteins interspersed within the plasma membrane [100]. These integrins are in direct communication with the extracellular matrix (ECM) proteins such as fibronectins, collagen, and laminins [101]. Conformation changes within the integrin domains and receptor clustering (larger α subunit and a smaller β subunit) lead to the engagement with ECM proteins [101,102]. This results in the formation of Focal Adhesion Complex (paxillin, talin, actinin, vinculin, and integrin αVβ3) and activation of focal adhesion kinase (FAK) [102,103,104]. FAK auto-phosphorylation causes binding with GRB2 (growth factor receptor bound protein 2) and phosphorylation of adaptor protein SHC [105]. This leads to the activation of downstream signaling molecules ranging from RAS (Rat sarcoma virus GTPases), RAF (Rapidly Accelerated Fibrosarcoma), MEK (Mitogen activated protein kinase), ERK, JNK (Jun N termina kinase), Rho (Ras homologous proteins), PAK (P21-activated protein kinases) and MLCK (Myosin Light Chain Kinase). These signaling events form the basis for physiological functions such as cytoskeletal alterations, gene transcription, contraction, cellular survival, proliferation, cell invasion, and cell migration (Figure 5) [102,105].

### 4.9. Half-Life and Clearance of GFAP

GFAP is synthesized and secreted by the astrocytes and circulates in the brain interstitial fluid and cerebrospinal fluid. Eventually it will cross the blood–brain barrier and enter the systemic circulation. Its half-life and clearance might vary depending on its location. According to Price, J.C. et al., protein circulating in the brain might have a significantly slower turnover (9 days) as compared to blood (3.5 days) [106]. In 20-day old mice injected with [guanido-14C] arginine, extraction of cytoskeletal filaments from the spinal cord indicated revealed slow turnover of GFAP and protracted persistence at 9 weeks [107]. Studies performed to assess the GFAP turnover rate in the astrocyte cultures revealed both monophasic as well as biphasic decay kinetics.

Analysis of the synthesis and turnover of GFAP in the primary astrocyte cultures of the rat indicated the presence of bi-phasic decay kinetics with the faster decaying pool having a half-life around 12–18 h and a slow decaying pool displaying a half-life of approximately 5–8 days [108,109]. Characterization of the turnover of GFAP in the astrocytes treated with growth factors revealed a half-life of 7.5 days [110]. In the clinical research performed to assess the serum markers in the TBI patients, the half-life of GFAP (24–48 h) was longer than UCH-1 [Ubiquitin carboxy-terminal hydrolase L1] thus making it one of the preferable markers for the acute and subacute phases of TBI patients [111]. In patients with mild to moderately severe TBI, the half-life of GFAP tend to fluctuate with 6–48 h in those who survived as compared to 61–84 h in those succumbed to severe complications [112].

Abnormal kinetics with respect to the synthesis and degradation of GFAP is implicated in the pathogenesis of AxD [113,114]. In AxD, synthesis and degradation of GFAP are equal at least in the initial stages [113]. Unfortunately in the later stages, there is a lag phase where degradation of GFAP does not match with the synthesis of a new protein, a change that paves the way for accumulation of GFAP in the brain tissues [113]. Finally in the equilibrium phase, degradation of protein slowly increases to keep up with the synthesis, nevertheless maintaining the status quo of higher levels of GFAP in the spinal cord and brain tissues. These will trigger the accumulation of Rosenthal fibers, neurodegeneration, and onset of disease manifestations [113].

## 5. TBI and GFAP

### 5.1. TBI and Its Correlation with AD

Serial follow-up of TBI patients revealed that they are more likely to be inclined to develop AD, chronic traumatic encephalopathy, and Parkinson’s disease (PD) [115,116,117]. TBI induces axonal injury, blood–brain barrier dysfunction, glial cell activation, and neuroinflammation, thus invigorating numerous signaling pathways for stockpiling of APP, alpha synuclein (α-syn), hyper-phosphorylated Tau, and TAR DNA-binding protein 43 (TDP-43) [116,117].

Numerous studies conducted so far have established a relationship between TBI injury and AD [118,119,120,121,122]. In a study by Mielke, M.M. et al., where they evaluated 1418 patients of 40 years or older by regular follow-up, TBI patients were 1.3-fold more likely to develop AD as compared to controls (age-, sex-, and body injury-matched) [123]. In a retrospective case-control study, where they analyzed 150 clinical cases of head injury in two geriatric clinics in Seattle, Washington, AD was more prevalent (OR-3.5) and was statistically significant (*p* = 0.0006) in TBI patients within a 5-year timeframe [124].

It would be worthwhile to understand the possible mechanisms that underlie development of AD in TBI patients. The occurrence of TBI seems to provoke a set of cellular changes including microglial activation, M1 phenotype predominance, and augmented neuroinflammation. This concoction of aberrations will ultimately trigger memory/learning abnormalities, reduced neuronal density, disorganized synapses, and exacerbated Aβ plaques in the hippocampus of APP/PS1 mice [125]. Mice subjected to TBI injury developed tau accumulation in the ipsilateral cortex, as well as in the amygdala, hippocampus, and brain stem of the contralateral cortex eventually within a week, thus kickstarting AD-like pathological changes [126]. This increased tau phosphorylation in TBI-injured mice can be explained due to acceleration/deceleration forces causing rapid collision of the ventral surface of brain with the posterior skull [126]. Appearance of tau lesions farther from the site of head injury might indicate propagation of these pathological aggregates via neuronal interconnections [126].

Alternatively, it has been speculated that multiple and repetitive head injuries might eventually give rise to dementia through chronic traumatic encephalopathy (CTE). TBI-induced axonal shearing causes separation of tau from tubulin and its subsequent phosphorylation; thereby, resulting in the formation of tau oligomers [116]. Eventually, NFT crystallization ensues from the aggregation of these tau oligomers [116]. These NFTs escape into the extracellular space, spread among neighboring neurons, thus facilitating the widespread propagation of tau pathology and abetting in disease progression [116]. Based on these studies, it is quite evident that TBI enkindles cumulative agglomeration of amyloid deposits and NFTs and thereby heightens the risk of developing AD over a period of time. Concurrently, TBI can derail the integrity of BBB, thus provoking inflammatory cell immigration from peripheral circulation as well as microglial/astrocyte activation within the brain tissues. Synchronously, tau-oligomers accumulation along with glial cell inflammatory outburst might predispose to the evolution of CTE [116].

### 5.2. Astrogliosis and TBI

There are few studies that interconnect the development of astrogliosis and its ramifications with the pathology of TBI. TBI-induced mechanical forces tend to initiate varying degrees of brain tissue damage along with interruption of the brain homeostasis [127]. This consequent neuronal cellular injury will set in motion an assortment of local cellular defenses to straighten out these neuronal fallibilities [127]. Astrogliosis can be considered as one of the most important primary cellular innate immune defenses for countering these neuronal damages as well as for kick-starting neuronal regeneration [127]. In this regard, it would be prudent to recognize that astrogliosis will be instrumental in advancing tissue repair, tempering inflammation, modulating the blood–brain barrier, and aiding synaptic plasticity and neuronal reorganization [127,128].

Studies indicate that astrogliosis can occur due to various mechanisms as TBI-induced brain injury emanates [127,128]. According to Joshua, J.E. et al., TBI can strain the astrocyte intermediate filament networks and arouse continuous activation of mechano-sensitive ion channels within its plasma membrane [127]. This will foster the constant entry of extracellular ions, thus sparking off hypercalcemia and ATP generation [127]. These cellular changes will form the deep-seated basis for invigoration of cellular pathways in an autocrine and paracrine manner, thus spawning the production of glutamate, MMP-9 [Matrix metalloproteinase-9] and biomarkers (GFAP and S100-B) [127]. These engendered by-products will thereby lead the way for proliferation and hypertrophy of astrocytes, thus giving rise to reactive astrocytes with defensive and tissue healing functions [127]. As per Ben-Gigi, L. et al., astrogliosis secondary to TBI is primarily dependent upon phosphorylation of SemaB825 primarly dependent upon Type 4 transmembrane protein, Semphorin in the astrocytes

(Type 4 transmembrane semaphorin) in the astrocytes [129].

**Figure 6:**
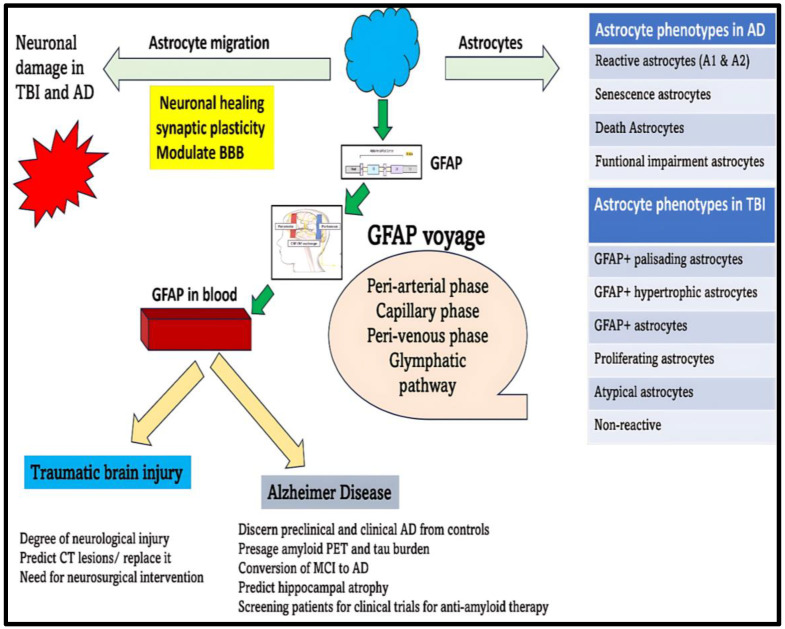
Clinical utility of GFAP as a biomarker in TBI and AD. Neuronal damage induced during TBI and AD pathogenesis will provoke astrocyte migration and morphological transformation. Studies speculate that there are different morphological phenotypes of astrocytes in TBI and AD. These multiple phenotypes of astrocytes will be instrumental in neuronal repair and healing. As a part of astrogliosis, there will be overproduction and concomitant release of GFAP into the neuronal extracellular space. GFAP will transverse through peri-arterial, capillary, peri-venous, and glymphatic pathways and will ultimately escape into the systemic circulation. The fluctuations of GFAP in the blood can be used as a premonition to forecast clinical severity and prognosis of TBI and AD. Unfortunately, due to TBI- and AD-induced encumbrances blocking the excretory pathway, smooth sailing of GFAP from brain circulation into systemic circulation should not be expected. Future research studies are needed to ascertain these impediments in the disease context before we can safely rely on GFAP for presaging disease-related changes in TBI and AD.

The reactive astrocytes are known to assume various morphological forms during TBI evolution to propagate numerous physiological and pathological functions. These reactive astrocytes can metamorphose into GFAP+ palisading astrocytes, GFAP+ hypertrophic astrocytes, GFAP+ astrocytes, proliferating astrocytes, atypical astrocytes, and non-reactive astrocytes (Figure 6) [130]. These transfigured reactive astrocytes are primarily instrumental in effectuating immune-modulation, neuro-modulation, and scar formation, all of which form the cardinal basis for tissue healing and neuroinflammatory actions in response to the TBI-induced neuronal injury [127]. According to Michinaga, S. et al., the pro-inflammatory environment in the backdrop of TBI will foment the building up of cytokines, particularly IL-1 and endothelin-1 (ET-1) [128]. These two cytokines will act upon their respective receptors on the astrocytes to enkindle the activation of nuclear transcription factors (STAT-3 [Signal transducer and activator of transcription 3], SP-1 [Specificity protein 1], and NF-KB [Nuclear factor kappa B]) [128]. On these grounds, there will be upregulation of GFAP, Cyclin-D1, and S-phase kinase-associated protein-2 (Sp-2) proteins. This combination of proteins will act together to set the ball rolling for dawning astrogliosis [128]. Unfortunately, the degree of astrogliosis does not unfold in a controlled manner, thus exacerbating the neuronal injury through instigating cytotoxic edema, BBB disruption, and neuroinflammation, thereby fanning the flames for amplifying and abetting TBI-induced neurodegeneration [127,128]. To support this hypothesis, mice subjected to repetitive mild TBI encountered a unorthodox astrocytic response characterized by atypical astrocytes, no scarring, lessened GFAP upregulation, impaired astrocytic coupling, and reduced expression of homeostatic proteins, all of which contributed to development of spontaneous recurrent seizures in mice [131].

In an animal study by Mathewson, A.J. et al., cerebral contusion in rats provoked enhanced upregulation of GFAP in the palisading astrocytes bordering the stab injury along the ipsilateral cortex [132]. As time progressed, this increased GFAP reverted back to normal levels in the cerebral cortex except in the zone of palisading astrocytes surrounding the healing cerebral tissue [132]. These findings highlight the fact that GFAP upregulation in the astrocytes will persevere as long as tissue healing process is complete as it might indirectly have a hand in the structural reorganization and recuperation of the healing brain tissues [132]. Given this cellular transpirations, upregulated GFAP levels are more likely to predict brain lesions on imaging studies and clinical prognosis as compared to S100B levels in TBI patients [133]. Despite these findings, the GFAP and S100B are not likely to be considered to be peripheral biomarkers in TBI due to their lower positive predictive value (PPV) [133].

### 5.3. GFAP and UCH-L1 (Ubiquitin C-Terminal Hydrolase-L1) Levels in TBI (Traumatic Brain Injury)

Due to the presence of active peri-vascular drainage and glymphatic clearance pathways, neuronal (UCH-L1) and glial (GFAP) proteins that are produced in the brain tend to abscond from the CSF into the systemic circulation. Due to this exit strategy, these emigrated proteins circulating in the plasma can reliably forecast information regarding the severity and prognosis of neurological damage in TBI [134]. UCH-L1 is protein which is widely expressed in the brain tissues [135]. According to the American Academy of Family Physicians, assaying GFAP and UCH-L1 in the plasma to predict severity of the neuronal injury following TBI might reduce the utilization of expensive imaging tests (CT scans and MRI [Magnetic Resonance Imaging]), a strategy that can be extremely useful in the primary care centers [136]. This particularly makes a dramatic difference in the rural hospitals where health care access is limited, and low insurance coverage is commonplace.

Numerous clinical research studies were conducted to compare the efficacy of GFAP and UCH-L1 in predicting the severity of TBI. In a multi-center observational study, plasma GFAP and UCH-L1 has higher sensitivity [0.976 (95% CI 0.931–0.995)] and negative predictive value (NPV) [0.996 (0.987–0.999)] in presaging the neuronal injury in TBI. Furthermore, a positive CT scan for TBI in case of a negative blood test for GFAP and UCH-L1 was evident in less than 1% of the cases [137]. Interestingly, UCH-L1 was preferentially elevated in the children with non-concussive trauma as compared to GFAP, thus raising the presumption that UCH-L1 is a non-specific marker of neuronal damage [138]. In parallel to these findings, UCH-L1 was also found to be a non-specific marker of neuronal damage in the murine models of TBI [139]. So, the authors in this study speculated that UCH-L1 is better suitable to be used as an adjunct marker in identifying central or global ischemia rather than a specific marker of neuronal injury in TBI [139]. After TBI, GFAP and UCH-L1 can be detected as early as 8–20 h and persisted until the 48 h–7 days’ timeframe [111]. It was shown that both UCH-L1 and GFAP were marginally effective in predicting mild–moderate TBI, CT lesions, and the need for neurosurgical intervention over a 7-day period following TBI [111,140]. Long term neurological outcomes of 6-months duration were also accurately predicted by these two markers [141,142]. In a multicenter prospective observational study examining young adults presenting to the emergency department, both GFAP and UCH-L1 were useful for clinicians in making decisions regarding a need for a CT scan to rule out potential intracranial hemorrhage [137,143].

Recently, the Federal Drug Administration (FDA) approved the Banyan Trauma Indicator (BTI) assay for streamlining the requirement of CT scans in mild to moderate TBI cases [144]. The sensitivity (97.5%) and specificity (99.6%) of this non-invasive blood test (GFAP and UCH-LI) are substantially high so that it can implemented in the hospital settings [144]. However, subsequent larger clinical trials are still the pressing, current need for validation of their cutoff values, diagnostic/prognostic algorithms, and neuroimaging parameters [145]. This will bestow safety for application of these markers in clinical settings for presaging neurological injury and prognostic outcomes in the TBI patients.

### 5.4. TBI Itself Might Derail the Release of Biomarkers into the Systemic Circulation

The reliability of these blood markers in forecasting the neurological outcomes depends on their fruitful emigration from the brain into the systemic circulation via para-vascular and lymphatic pathways [146]. Inhibition of glymphatic clearance by sleep deprivation, acetazolamide therapy, use of barbiturates, cisterostomy, and ventriculostomy (altering CSF production) can thwart the normal drainage of solutes and serum markers following TBI [146,147]. Increased intracranial pressure associated with TBI injury is shown to be associated with aberrations in the meningeal lymphatic system and lymphatic system [147,148]. Therefore, this will result in less efficient and downgraded circulation ultimately leading to dwindling interstitial fluid [ISF] and CSF turnover out of the brain vasculature [147,148]. Furthermore, specific morphological abnormalities in the lymphatics unmasked during TBI including increased capillary loops and sprouts/dilated lymphatic capillaries which might be responsible for diminished seepage of these circulating biomarkers from the brain circulation [148]. In mice subjected to TBI, at least a 60% reduction in the lymphatic drainage was the prelude for piling up of toxic aggregates within the brain tissues, which subsequently triggered tau aggregation and neurodegeneration [149]. All these above lymphatic drainage aberrations seemed to persist at least 1–2 months post-TBI injury, thus thwarting the efficient drainage of Aβ, tau, and other biomarkers such as GFAP and UCH-L1 into the systemic circulation [147,148,149]. It is logical to conclude that these above-mentioned tissue changes might crash the chances of rise in the blood markers immediately after TBI and thus precluding them from being useful for surveillance of neurological damage, disease severity, prognosis, and clinical outcomes in the TBI.

## 6. AD and GFAP

### 6.1. Astrocytic Damage in AD

The neurodegenerative process in AD elicits various metabolic and morphologic changes in the astrocytes which can ultimately backfire to accentuate the progression of disease process. These intracellular changes in astrocytes include impaired glutamate receptor uptake capacity, mitochondrial damage, altered cholesterol metabolism, and metabolic deficiencies [150,151]. Microglial activation associated with AD can drive the astrocyte-induced production of toxic free fatty acids and long-chain saturated fats, [150,152]. Moreover, active inflammatory markers such as IFN-gamma, IL-1, and LPS can energize the astrocytes to generate nitric oxide, oxygen radicals, and reactive oxygen species (ROS), as well as pro-inflammatory cytokines [150,153]. Hence, metabolic reprogramming and neurocytotoxic transformation of astrocytes during neurodegeneration can precipitate the neuronal demise and hasten disease progression.

According to Kim, J. et al., the pathological phenotypes of astrocytes encountered in AD disease models include reactive phenotypes (A1 and A2) and death phenotypes (apoptotic phenotype and ferroptotic phenotype, senescence phenotype, and functional impairment phenotype) (Figure 6) [26].

### 6.2. GFAP as a Reliable Biomarker in Diagnosis and Prognosis of AD

GFAP-expressing astrocytes can demonstrate decreased glutamate transport activity and uptake in the frontal cortex of AD, thus resulting in accumulation of glutamate in the synaptic clefts [26,154]. Accumulated glutamate stimulates NMDA [N-methyl-D-aspartate] receptors in the neurons, thus inciting oxidative stress, mitochondrial dysfunction, and caspase-3 activation in the AD brain [154,155]. This caspase-3 generated might cleave the tau aggregates particularly at Asp421 [Aspartate 21], thus resulting in formation of caspase-cleaved tau [156]. Their procreation might be a triggering point for the inception of early MCI pathology in AD disease process [156].

**Figure 7:**
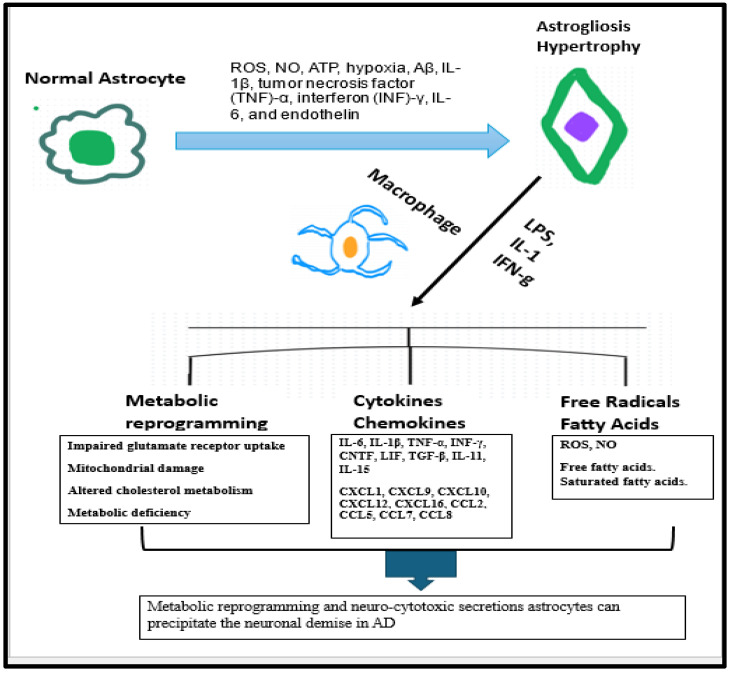
Astrogliosis and its repercussions that trigger neuronal demise.

The neurodegenerative process in AD elicits various metabolic and morphologic changes in the astrocytes which can ultimately backfire to accentuate the progression of disease process (Figure 7). The intracellular changes in astrocytes include impaired glutamate receptor uptake capacity, mitochondrial damage, altered cholesterol metabolism, and metabolic deficiencies [150,151]. Microglial activation associated with AD can drive the astrocyte-induced production of toxic free fatty acids and long-chain saturated fats, [150,152]. Moreover, active inflammatory markers such as IFN-gamma, IL-1, and LPS can energize the astrocytes to generate nitric oxide, oxygen radicals, and reactive oxygen species (ROS), as well as pro-inflammatory cytokines [150,153]. Hence, metabolic reprogramming and neuro-cytotoxic cytokine profile of astrocytes during neurodegeneration can precipitate the neuronal demise and hasten disease progression. Amassment of Aβ deposits in the brain can inflict astrocyte death, which can ultimately precipitate the seepage of GFAP from astrocytes into the neuronal extracellular milieu [157]. Due to this transpiration, GFAP released extracellularly can be a potential marker for diagnosis and prognosis in the AD disease continuum.

Due to this transpiration, Aβ+ participants were shown to have higher blood GFAP levels as compared to Aβ− participants [157]. In addition, plasma GFAP and Aβ _(1-42/1-40)_ demonstrated positive and negative correlation with medial temporal lobe atrophy and were beneficial in accurate forecasting of amyloid Positron emission tomography [PET] burden in a recently conducted clinical trial [158]. In The Translational Biomarkers in Aging and Dementia (TRIAD) study, plasma GFAP levels were precise in capturing the brain pathology so that it was possible to discern between preclinical AD, clinical AD, and cognitively unimpaired Aβ-negative individuals [159]. In preclinical AD, the plasma levels of GFAP were 285.0 [142.6] pg/mL and 185.1 [93.5] pg/mL in the in Aβ positive and Aβ negative cohorts, respectively, in the TRIAD study [159]. In AD continuum, levels of plasma GFAP were 285.0 [142.6] pg/mL, 332.5 [153.6] pg/mL, 388.1 [152.8] pg/mL, and 185.1 [93.5] pg/mL in cognitive unimpaired Aβ positive cohorts, MCI, AD, and Aβ negative cognitively normal cohorts, respectively [159]. Another important finding discovered in this study is that plasma GFAP (AUC: 0.69–0.89) was highly superior in discerning the Aβ positive from Aβ negative cohorts as compared to the CSF GFAP (AUC: 0.59–0.76) [159].

#### S100 Implication in AD

A few research studies have indicated that S100 levels are increased

Studies suggest that GFAP is elevated significantly more than S100B in the CSF of AD patients as compared to Creutzfeldt-Jakob disease (CJD) and control patients [160]. On the contrary, S100B is higher in the CSF of Creutzfeldt-Jakob disease (CJD) patients as compared to AD and control patients [160].

According to fee research studies, S100 levels are increased in AD patients as compared to control cohorts. This might be partially intertwined with their role in the digestion of amyloid precursor protein, as well as reinforcing behavior in facilitating Aβ deposition and tau phosphorylation [161]. Moreover, their frequent co-localization with Aβ aggregates in the AD brain might signify their underlying role in potentiating Aβ aggregation in AD [161]. Furthermore, their contribution on the furtherance of AD pathogenesis stems from their ability to modify AD signaling pathways, trace metal homeostasis, and cytokine metabolism [161].

Another important clinical end point which neurologists would be interested to glean is the time at which MCI cohorts undergo transmutation into full blown AD dementia for treatment justification. Forecasting this specific time-point is crucial because thorough management including careful monitoring, frequent CT/MRI scans, anti-amyloid medications, and enrollment in clinical trials can be tailored for better clinical outcomes. According to AD association, the FDA recently approved medications (Aducanumab and Lecanemab) were potent in changing the clinical progression in early AD as well as alleviating symptoms in moderate to severe AD (memantine) [162,163,164,165]. In this regard, recent studies have demonstrated that plasma GFAP is a non-invasive tool to better capture the time points at which Aβ positive and MCI cohorts transform into AD dementia [166,167]. Plasma GFAP levels in MCI-AD cohorts were 300 pg/mL, IQR 232–433 pg/mL, and *p* < 0.0001, and these levels increased to 360 pg/mL, IQR 253–414 pg/mL, and *p* < 0.01 in MCI-AD cohorts developing dementia during 3.9 ± 2.6 years follow-up period [168]. In parallel with these findings, higher blood GFAP levels (>232 pg/mL) were associated with greater attrition of hippocampal volume and increased risk of developing cognitive deficits in the elderly population [168]. Moreover, higher plasma GFAP was more likely correlated with a substantial reduction in the white matter volume in temporal and parietal lobes of the MCI and AD disease cohorts [169].

### 6.3. Does GFAP Circumnavigate Seamlessly in AD Brain? Fallacies in Excretory Pathways

After its release from the activated astrocytes, GFAP has the inherent ability to abscond from the brain circulation due to AD-driven BBB mutilation and its seepage through robust excretory pathways [170,171,172]. In the healthy population, the glymphatic excretory is divided into three phases, namely peri-arterial phase, capillary phase, and venous phase [171]. Along with other solutes, GFAP will navigate through each of these compartments to depart from the brain circulation. According to Salvado, G. et al., piling up of toxic Aβ during early stages of AD instigates the breakdown of the BBB and this will spur the direct release of GFAP from astrocytic end feet which are closely abutted to the basement membrane of the vasculature forming the BBB [173]. As a result, GFAP enters into the blood and thus can be non-invasively assayed with the help of state-of-the-art single-molecule array (Simoa) assays [174].

Unfortunately, the trickling of GFAP from the brain circulation to the systemic vascular vessels in the AD is not always unhampered due to the peculiar set of circumstances prevailing in the glymphatic circulation, cerebral vasculature, and blood–brain barrier. Low AQP4 [Aquaporin Q4] expression, migration of AQP4 from the astrocytic end feet, attenuated pulsations of arterial vessels, accumulation of Aβ in the glymphatic space, and sleep apnea are some of underlying aberrations in the AD that are implicated to perturb the smooth sailing of GFAP emigration out of the brain circulation [43,172]. On the account of these digressions, the excursion of GFAP from its origin to the systemic circulation might be halted.

Moreover, AD disease pathology-induced damage of the blood–brain barrier cripples the CSF drainage, thus unleashing collateral effects on the GFAP decampment from the brain circulation into the blood. The BBB is dysfunctional in AD secondary to various pathological aberrations ranging from oxidative damage, neuroinflammation, altered metallo-proteinases, faulty amino-acid transporters, ionophore formation, and dysregulated LRP1 [Low-density lipoprotein-related protein 1]/RAGE [Receptor for advanced glycosylation end products] transport [170,175]. Conceivably, toxic aggregates of Aβ heaping up in the perivascular pathways might clog the microvasculature, thereby restraining the smoother passage of GFAP out of the brain circulation [171].

Oxidative stress and inflammatory markers will facilitate the transmutation of physiological astrocytes into hypertrophic astrocytes, thus opening the doors for inception of astrogliosis. Although this transformation is initially protective, it will be counterproductive due to the influence of activated microglial cells and pro-inflammatory factors such as metabolic reprogramming, cytokines, chemokines, and free radicals. 

Under the influence of surrounding energized microglial cells as well of concomitant production of cytokinesm chemokines and free radicals.

Therefore, metabolic reprogramming and inflammatory secretions of astrocytes will perpetuate the neurodegeneration in AD.

### 6.4. FDA-Approved Assay for the AD Diagnostics Focusing on Spatiotemporal GFAP Profiles in AD and TBI

FDA recently approved a non-invasive plasma test (p-tau217/Aβ42 ratio) [Phospho-tau 217/Amyloid beta42] that can accurately predict the amyloid and tau burden in the brain of clinical and community cohorts [176,177]. This blood test outperformed CSF p-tau181/Aβ42 [Phospho-tau 181/Amyloid beta42] and Aβ42/Aβ40 [Amyloid beta42/Amyloid beta 40] and blood p-tau217, Aβ42/Aβ40, p-tau181, and p-tau181/Aβ42 in the predicting the Aβ and tau PET pathologies in the clinical and community cohorts [176,177]. Lumipulse G pTau217/ß-Amyloid 1-42 plasma ratio has not been cleared by FDA for traumatic brain injury. FDA has approved an i-STAT TBI test cartridge test, which is a non-invasive blood test for the diagnosis of TBI injury [136]. In this test, a small amount of blood is applied to the i-STAT cartridge which is then used to measure the release of GFAP and UCH L1 into the peripheral blood stream within 24 h of traumatic brain injury [136].

## 7. Summary Points

(a) Astrogliosis fomented fingerprint, GFAP, is a 50 kDa cytoskeletal protein whose gene can be traced to the chromosome 17.
(b) From a structural standpoint, it is comprised of a head domain, rod domain, and tail domain.
(c) The filamentous form of GFAP is influenced by the aggregation of individual monomers into dimers and tetramers.
(d) Physiologically, there exists a dynamic equilibrium between assembly and disassembly of individual subunits.
(e) GFAP participates in various physiological functions including having a trophic effect on neurons, BBB maintenance, myelination, synaptic plasticity, glutamine transport, and neuronal growth.
(f) GFAP tends to exist in many isoforms. The principal isoform responsible for fostering many physiological functions in the brain is GFAP-α.
(g) Under pathological conditions, GFAP mRNA can be jolted to endure alternative splicing, and this native mRNA eventually procreates to give birth to different isoforms namely GFAP-β, GFAP-γ, GFAP-δ, GFAP-κ, and GFAP-ζ.
(h) The stability of GFAP filament is regulated by four types of post-translational modifications namely phosphorylation, citrullination, acetylation, and hyper-palmitoylation.
(i) Unfortunately, GFAP (50 kDa) is assailable and vulnerable to degradation by calpain and caspases resulting in bringing forth petite fragments known as breakdown products (BDPs) with a lower molecular weight ranging from 26 to 44 kDa. These BDPs can fall outside of detection range and sometimes evade detection by standard antibody detection kits.
(j) GFAP direct cytoskeletal interactions mainly with plectins and actin microfilaments foster indirect communications with integrins and extracellular matrix proteins. These interactions are decisive in executing cardinal cellular functions such as survival, proliferation, invasion, and migration.
(k) GFAP can be a future blood-based biomarker that can reliably forecast brain damage and reduce the need for CT scans for diagnosis and prognosis of TBI, a factor making a huge difference in primary care centers (Figure 6).
(l) GFAP can also perform as an excellent marker for auguring the diagnosis of preclinical AD, stockpiling of toxic aggregates, as well as disease progression in AD (Figure 6).
(m) The potency of GFAP to function as a trustworthy marker in blood is primarily dependent on its successful escape from the brain tissues through the peri-arterial, peri-venous, and glymphatic pathways into the systemic circulation. Unfortunately, various quagmires due to TBI and AD, which might be operational, can curb its smooth transport into the blood. Understanding these potential fallibilities is essential before we relate the fluctuations of GFAP in the blood to disease changes in TBI and AD.

## 8. Future Directions

Although blood GFAP seems like a viable alternative for capturing the brain changes for forecasting disease progression, there is still a lot of endeavor that needs to be undertaken to cement its potential as a trustworthy marker in AD. Moreover, further research is also warranted on the various GFAP isoforms and their relative significance in relaying various AD disease stages. It is also plausible that these GFAP isoforms might undergo isotype switching to presage the disease status as AD marches through progressive BRAAK stages. Future researchers should be cognizant of its post-translational modifications and its cleavage pattern as it might cloud its detection in the body fluids.

The clinical performance of GFAP in serum and blood in different clinical contexts and age cohorts should be thoroughly investigated in multi-centered clinical trials [178]. This will provide us with clinical applicable assays that can be validated and used in the hospital settings for differentiating AD- and TBI-induced tau pathology [178]. Furthermore, studies should be conducted to ascertain the pattern of GFAP escape from the brain circulation into blood in AD and TBI [178]. The pattern of BBB injury in TBI and AD and its influence GFAP immigration should be delved into so that we would interpret diagnostic tests with better sensitivity and specificity. As plasma GFAP better correlates with amyloid and tau pathology than CSF GFAP, it would be ideal to investigate the underlying physiology for better performance of blood GFAP vs. CSF GFAP [179]. Moreover, the effect of BBB dysfunction and impaired glymphatic clearance pathways on the migration of GFAP from brain circulation to systemic circulation should be quantified so that we can accurately validate its immunoassays for detection.

Furthermore, we surmise that the reliability of these blood-based markers such as GFAP should be temporarily paused until we carefully appraise the extent of wreckage in the glymphatic excretory pathways and their ensuing impact in undermining the active hauling of these astroglial markers safely into the systemic circulation in TBI and AD. Although some of the clinical studies previously executed provide us with enough evidence to incorporate plasma GFAP as non-invasive monitoring biomarker for diagnosis and prognosis of AD in the clinic, there is still a need for validating clinical trials. These clinical trials should be conducted in multiple centers, and they should define appropriate protocols, specific cut off points, and specific recommendations to be followed for accurately quantifying these biomarkers. Therefore, pending further clinical research studies that sheds light into these structural aberrations and derailment of drainage pathways, deciphering the fluctuations of GFAP in AD and TBI needs to be taken into cognizance with careful forethought.

## 9. Limitations

This review has number of limitations. First, only a PubMed search was used in this review. Other databases were not utilized to interpret the GFAP biology and its effectiveness as a biomarker in TBI and AD. Second, the accumulation of GFAP in the blood and its reliability as a biomarker might ultimately depend upon the presence of absence of these fallacies including BBB devastation. Furthermore, PTMs, isoforms, and conformational dynamics of GFAP might influence its detection and might give rise to differential diagnostic detection in AD and TBI [83]. Moreover, astrogliosis and GFAP upregulation is not specific for AD dementia and any neurological injury from fronto-temporal dementia or vascular dementia can evoke astrocytic response and GFAP upregulation. Appropriate quantification of GFAP levels in different forms of dementia would be worthwhile.

## Data Availability

Not Applicable.
